# HIV-1 infected patients with suppressed plasma viremia on treatment have pro-inflammatory HDL

**DOI:** 10.1186/1476-511X-10-35

**Published:** 2011-02-23

**Authors:** Theodoros Kelesidis, Otto O Yang, Judith S Currier, Kaveh Navab, Alan M Fogelman, Mohamad Navab

**Affiliations:** 1 Division of Infectious Diseases, Department of Medicine, David Geffen School of Medicine, University of California, Los Angeles, CA, 90095, USA; 2 Department of Microbiology, Immunology, and Molecular Genetics, David Geffen School of Medicine, University of California, Los Angeles, CA, 90095, USA; 3 Division of Cardiology, Department of Medicine, David Geffen School of Medicine, University of California, Los Angeles, CA, 90095, USA

## Abstract

**Background:**

The role of pro-inflammatory lipids in systemic immune activation in HIV infection remains largely unknown. We hypothesized that HIV-1-infected persons on antiretroviral therapy would have pro-inflammatory high density lipoprotein (HDL), and that an apoA-1 mimetic peptide might reverse the inflammatory properties of HDL in these persons.

**Methods:**

Plasma was obtained from 10 HIV-1-infected individuals on combination antiretroviral therapy with suppressed viremia and was incubated with the apoA-I mimetic peptide L-4F or sham-treated prior to isolation of HDL. The HDL that was isolated from each sample was tested for its ability to inhibit LDL-induced MCP-1 production in cultures of human aortic endothelial cells.

**Results:**

We found in a small pilot study of HIV-1-infected individuals with suppressed viremia on combination antiretroviral therapy that oxidative stress and inflammation in HIV-1 are associated with a marked reduction of HDL antioxidant/anti-inflammatory activities. In vitro, these abnormalities were significantly improved by treatment with the apoA-1 mimetic peptide, 4F.

**Conclusions:**

These preliminary observations suggest that the anti-inflammatory properties of HDL are defective in HIV-1-infected persons despite treatment that is considered to be virologically successful.

## Findings

With improved antiretroviral therapies and survival among patients with HIV-1, cardiovascular disease (CVD) has become an increasingly important cause of morbidity and mortality [1]. The magnitude of the increased CVD risk among persons with HIV-1 infection is unclear, however, as are the relative contributions of viremia, immune activation, antiretroviral therapy (ART), conventional cardiovascular risk factors (such as increasing age and hypertension), and changes in systemic inflammation [1]. Understanding the relative contributions of host, virus, and antiretroviral therapy to risk of CVD in HIV-1 infection will aid in the development of strategies for prevention and treatment.

Inflammation has increasingly been recognized to be pivotal in the initiation and perpetuation of arterial injury leading to atherosclerosis and its complications [2]. Pro-inflammatory high density lipoprotein (HDL) may play a role in this process; higher serum levels of pro-inflammatory HDL are associated with the increased rates of CVD in certain chronic inflammatory conditions such as rheumatoid arthritis, systemic lupus erythematosus, and type II diabetes mellitus [3]. While immune activation is a hallmark of HIV-1 infection, it is not known if HIV-1 infection affects levels of pro-inflammatory HDL, or if increases in HDL cholesterol (HDL-C) observed after initiation of ART in longitudinal studies are neutral, atheroprotective or atherogenic in nature [2,4-8].

With regards to pro-inflammatory lipids, apoA-1 mimetic peptides have been shown to remove oxidation products from lipoproteins and cell membranes and restore structure and function of low density lipoprotein (LDL) and HDL in a wide range of experimental inflammatory conditions in animals [9]. In view of the systemic immune activation that is not entirely reversed despite effective ART [9,10] and which could therefore be conducive to generating pro-inflammatory lipids, we hypothesized that HIV-1-infected persons on ART would have pro-inflammatory HDL, and that an apoA-1 mimetic peptide might reverse the inflammatory properties of HDL in these persons.

## Materials and methods

### Patients

To test this hypothesis, plasma was obtained from 10 HIV-1-infected individuals on combination antiretroviral therapy with suppressed viremia (below 50 copies of RNA/ml). Informed consent was obtained from all subjects.

### Determination of proinflammatory HDL

Plasma was incubated with the apoA-I mimetic peptide L-4F at a concentration of 1 μg/mL for 15 minutes at 37°C, or sham-treated prior to FPLC fractionation. HDL-containing FPLC fractions were tested for their ability to inhibit LDL-induced MCP-1 production in cultures of human aortic endothelial cells as previously described [9]. Results were normalized as a ratio to the effect of control LDL without HDL to yield the HDL inflammatory index (HII). More specifically HII is calculated from the ratio LDL-induced monocyte chemotactic activity (as determined by MCP-1 production in cultures of human aortic endothelial cells) in the presence of HDL/LDL-induced monocyte chemotactic activity in cultures of human aortic endothelial cells in the absence of HDL [9]. Data were analyzed with Student's t-test using SPSS 13.5 software (Chicago, IL, USA) and expressed as mean ± SD; P-values less than 0.05 were considered significant.

## Results

**Figure 1:**
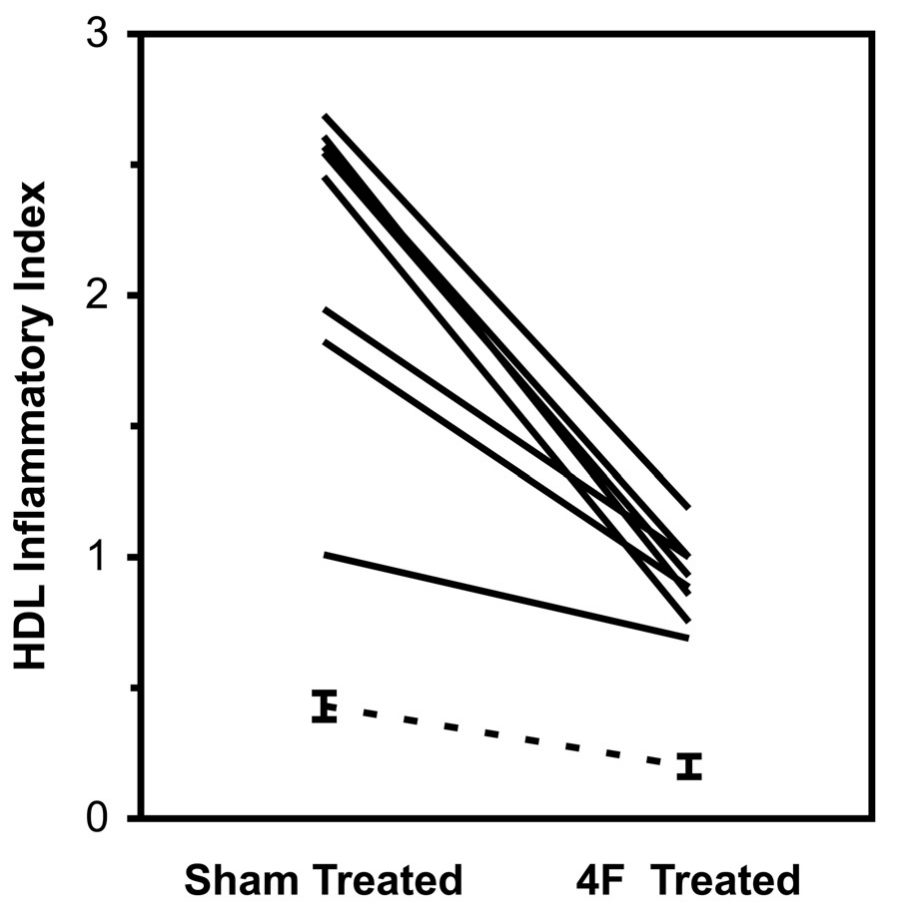
**HDL inflammatory index from plasma of patients with HIV (sham-treated versus L-4F- treated)**. The dotted line indicates previously reported values from healthy controls (0.43 ± 0.05 and 0.20 ± 0.04 for sham and 4F treatment respectively, taken from reference 4). In this assay, anti-inflammatory HDL is defined as HDL inflammatory index values < 1.0 and proinflammatory HDL is defined as HDL inflammatory index values > 1.0.

All subjects were men with an average age of 43 ± 14 years (± SD, range 21 - 63) on combination ART. Viral load and CD4^+ ^T cell counts were available on nine subjects (Table 1); these had plasma HIV-1 RNA values < 50 copies/mL and mean blood CD4^+ ^T cell counts of 595 ± 166 cells/mm^3 ^(range 367 - 966). Mean HII values for HDL in the sham treated plasma were 2.16 ± 0.63 (range 1.01 - 2.73) while mean HII values for HDL in the L-4F treated plasma were significantly lower (p < 0.0001) at 0.89 ± 0.16 (range 0.64 - 1.19). These values were compared to previously reported mean values from healthy controls (0.43 ± 0.05 and 0.20 ± 0.04 for sham and 4F treatment respectively (Figure 1) [9]. Thus, high-density lipoprotein isolated from plasma of persons with ART treated HIV-1 infection was highly pro-inflammatory, and was markedly less pro-inflammatory after treatment of plasma with 4F *in vitro *(Figure 1). Both the high baseline HII and reduction of the inflammatory index after 4F treatment were observed in all 10 samples, suggesting an overall pro-inflammatory lipid profile that was at least partially reversible by 4F.

**Table 1:** Characteristics of the HIV-1-infected study participants

Subject	Age	Sex	CD4 count (cells/mm^3^)	VL (RNA/ml)	TC (mg/dl)	HDL (mg/dl)	LDL (mg/dl)	TG (mg/dl)	HII (sham)	HII (L-4F)	ART
1	25	M	367	< 50	135	51	66	91	2.45	0.75	AZV, RTV, TDF, FTC
2	NA	M	NA	NA	NA	NA	NA	NA	2.69	1.19	NA
3	57	M	499	< 50	159	31	77	254	1.01	0.69	EFV, ABC, 3TC
4	44	M	540	< 50	NA	NA	NA	NA	2.73	0.96	TDF, FTC, RTV, FPV
5	44	M	540	< 50	286	26	212	240	2.55	0.93	TDF, FTC, AZV, RTV
6	46	M	571	< 50	184	52	108	121	1.82	0.89	NA
7	47	M	966	< 50	176	52	122	100	2.61	0.86	TDF, FTC, NVP
8	31	M	553	< 50	205	76*	109	98	1.24	0.64	EFV, FTC, TDF
9	21	M	712	< 50	169	64*	93	58	1.95	1.00	EFV, FTC, TDF
10	62	M	605	< 50	167	44	88	176	2.57	1.00	LPV, RTV, 3TC ETV

Abbreviations: AZV: Atazanavir; ABC: Abacavir; ART: Antiretroviral therapy; ETV: Etravirine; Efavirenz (EFV), FTC: Emtricitabine, FPV: Fosamprenavir; HDL: High Density Lipoprotein; HII; HDL Inflammatory Index; 3TC: Lamivudine; LPV: lopinavir; LDL: Low Density Lipoprotein; M: Male; NVP: Nevirapine; NA: Not available; TDF: Tenofovir; TC: Total Cholesterol; TG: Triglycerides; RTV: Ritonavir; VL: Viral Load

*:Samples were collected when patients were non-fasting.

## Discussion

Classically, HDL is believed to play an important role in mitigating oxidative stress and inflammation by uptake, processing and disposal of oxidized lipids [2,11]. However, systemic inflammation has been shown to lower this antioxidant and anti-inflammatory activity by transforming HDL to a pro-oxidant, pro-inflammatory acute-phase HDL that enhances the tendency of LDL to induce monocyte chemotaxis [2]. It has been demonstrated that mice that are genetically susceptible to develop atherosclerosis have augmented pro-inflammatory HDL [9,11]. HDL, therefore, not only removes excess LDL-derived cholesterol from peripheral tissues, but also has a major role in mitigating LDL-induced inflammation.

In our small cohort, all HIV-1-infected persons showed a marked reduction of HDL anti-inflammatory activity as evidenced by impaired HDL mediated inhibition of LDL-induced monocyte chemotactic activity. The observed defect cannot be attributed to low plasma HDL level in these patients, as equal amounts of isolated HDL were assayed; thus the observed HDL dysfunction must be due primarily to qualitative abnormalities. Oxidative stress and inflammation are nearly constant features of HIV-1 infection and the prevailing inflammatory state likely contributes to the observed reduction of HDL anti-inflammatory function in this population. This could lead to a vicious cycle in which the underlying inflammation and oxidative stress induce HDL dysfunction, which results in further inflammation.

Treatment of plasma samples from the HIV-1-infected subjects with the potent apoA-1 mimetic peptide 4F results in a marked increase in HDL anti-inflammatory activity. 4F is an 18-amino acid apoA-1 mimetic peptide, and its anti-inflammatory effects are mediated by its ability to preferentially remove oxidation products from lipoproteins and cell membranes, thereby restoring HDL and LDL function and structure, improving cell function, and attenuating inflammation [11]. ApoA-1 mimetic peptides have been shown to reduce atherosclerosis and attenuate inflammation in experimental animals without significantly changing plasma lipid levels [11]. 4F treatment in this study generally failed to return the pro-inflammatory activity of HDL to the low levels that we have previously reported in healthy controls [9].

These preliminary observations suggest that the anti-inflammatory properties of HDL are defective in HIV-1-infected persons despite treatment that is considered to be virologically successful, and that standard clinical lipid profile testing may not be an adequate measurement of the risk for CVD in these individuals. In this small pilot study we did not assess the possible association of proinflammatory HDL with other markers of immune activation and inflammation and the possible effect of 4F on these markers. An ongoing prospective study will aim at addressing the possible association of proinflammatory HDL with other markers of inflammation, immune activation and progression of atherosclerosis in patients with HIV infection. This study will also evaluate the effect of different types of antiretroviral treatment regimens versus HIV infection itself on the HDL inflammatory index.

In conclusion, to our knowledge this is the first demonstration that HIV-1 infection is associated with a marked reduction of HDL antioxidant/anti-inflammatory activities. In vitro, these abnormalities were significantly improved by treatment with the apoA-1 mimetic peptide, 4F.

## Abbreviations used

ART: antiretroviral therapy; apoA-1: apolipoprotein A-1; CVD: cardiovascular disease; FPLC: Fast protein liquid chromatography; HDL: High Density Lipoprotein; HIV-1: Human Immunodeficiency virus-1; HII: HDL inflammatory index; LDL: Low Density Lipoprotein; MCP-1: Monocyte chemotactic protein-1

## Competing interests

AMF and MN are principals in Bruin Pharma and AMF is an officer in Bruin Pharma.

## Authors' contributions

All authors contributed to the intellectual development of this work, and approved the final manuscript. TK, OY, KN and MN analyzed the data. TK searched the literature and wrote the draft paper. JC and AFM provided critical corrections to the manuscript.
